# Alcohol-induced deaths in the United States across age, race, gender, geography, and the COVID-19 pandemic

**DOI:** 10.1371/journal.pgph.0004623

**Published:** 2025-09-17

**Authors:** Tony Wong, Lucas Böttcher, Tom Chou, Maria R. D’Orsogna

**Affiliations:** 1 Department of Mathematics, University of California, Los Angeles, California, United States of America; 2 Department of Computational Science and Philosophy, Frankfurt School of Finance and Management, Frankfurt am Main, Germany; 3 Laboratory for Systems Medicine, University of Florida, Gainesville, Florida, United States of America; 4 Department of Computational Medicine, University of California, Los Angeles, California, United States of America; 5 Department of Mathematics, California State University at Northridge, Los Angeles, California, United States of America; The Ohio State University, UNITED STATES OF AMERICA

## Abstract

We analyze alcohol-induced deaths by race, gender, age and geography on a yearly (1999–2024) and monthly (2018–2024) basis, using data from the National Vital Statistics System. Crude rates for alcohol-induced deaths increased by 89% from 1999 to 2024. The largest relative increase occurred among females aged 25–34, with a 255% increase, and males aged 25–34, with a 188% increase. American Indian and Alaska Native populations remain the most affected. While alcohol-induced deaths are higher among males, crude rates are rising faster among females across all demographics, a concerning trend. Sharp increases occurred at the onset of COVID-19, peaking in 2021. For most demographics across the nation, crude rates remained abnormally high throughout 2023; significant decreases emerged only in 2024, four years after the start of COVID-19. Females were more impacted by alcohol-related liver disease than males; alcohol-related mental and behavioral disorders affected both genders. The largest monthly increases in alcohol-induced deaths occurred in American Indian and Alaska Native males (41% increase between May and June 2020) and females (32% increase between June and July 2020), Black females (32% increase between April and May 2020), males aged 15–34 (28% increase between April and May 2020) and females aged 35–44 (28% increase between April and May 2020). Since 2010, the highest crude rates have been in New Mexico. Record increases occurred in all states between 2019 and 2021; the largest was in Mississippi (122% increase between 2019 and 2021). By 2024, rates had returned within 10% of their 2019 levels in about half the states. In Oglala Lakota County (SD), McKinley County (NM), and Apache County (AZ), crude rates have exceeded an astonishing 80 fatalities per 100,000 annually since 2020. These findings emphasize the urgent need for targeted policies to reduce excessive alcohol consumption and improve access to treatment.

## 1. Introduction

Alcohol use disorder (AUD) is a medical condition characterized by the inability to limit alcohol use despite adverse physical or psychological consequences. Its diagnosis is based on established criteria [1] and is classified along a spectrum ranging from mild to severe. Typical indicators include drinking more or longer than intended, unsuccessful attempts to reduce alcohol use, and strong cravings for alcohol. Excessive alcohol consumption causes severe complications, such as alcoholic liver disease (ALD) [2–5], and contributes to metabolic syndrome, an ensemble of co-occurring conditions including arterial hypertension, diabetes, obesity, and other diseases [6–9]. Chronic alcohol-related conditions such as cirrhosis, alcohol hepatitis, strokes and heart failure may lead to death [10]. Ingesting large amounts of alcohol in a short period of time can also be fatal, as high blood alcohol levels may lead to respiratory failure. In some cases, alcohol consumption can engender suicidal impulses or impair judgment, increasing the risk of fatal accidents [11]. Alcohol withdrawal syndrome, which can also be lethal, is triggered by abrupt cessation of heavy drinking. AUD affects both genders [8,9,12], and has been exacerbated by the increasing popularity of binge drinking [13–15] and the practice of mixing alcohol with medications or illicit substances [16]. Even moderate consumption can be a risk factor for death in older adults with health issues [17].

Several long-term studies have tracked the evolution of both partially and fully alcohol-attributable deaths in the United States. These studies show that alcohol-related mortality has been rising over the past two decades [18–22]. Particularly concerning are the increases observed between 2019 and 2021, concurrent with the onset of the COVID-19 pandemic, when alcohol-related deaths displayed high increases compared to other causes of death [23–25]. Possible driving factors include higher alcohol consumption due to pandemic-related stressors and isolation, disruption to treatment programs, and difficulties accessing emergency facilities [26–31], similar to what was observed for increases in drug overdose deaths during the same period [32]. Most studies on alcohol-related mortality aggregate deaths without providing detailed analyses of the gender, age, geographical, and racial breakdown of fatalities. Understanding mortality trends across different population subgroups, however, is crucial for planning interventions and effectively allocating resources. Other critical questions are whether alcohol-induced deaths have returned to their pre-pandemic levels, and how post-pandemic alcohol-induced mortality differs among demographic groups or geographic locations. Answers to these questions can help determine which policies, if any, have been effective in reducing alcohol-related deaths. The aim of this paper is to provide an in-depth analysis of gender, age, geographical, and racial differences in fully alcohol-attributable deaths in the United States between 1999 and 2024, with special emphasis on the post-COVID-19 era. We focus on fully attributable alcohol deaths and do not consider deaths that are partially linked to alcohol consumption to avoid including other confounding factors, possibly pandemic-induced. Our data source and statistical methods are described in the Materials and methods section. In the Results section, we present our findings at the national, state and county levels, discussing stratifications by gender, age, race, cause of death. Finally, in the Discussion and in Conclusion sections we describe limitations of the current study and offer a future outlook.

## 2. Materials and methods

### 2.1. Ethics statement

The authors of this manuscript had no information that could identify individual participants during or after data collection. Provisional data for 2024 and finalized data for all other years were downloaded from the CDC WONDER database on January 28, 2025.

### 2.2. Data

This study utilizes mortality data from the Centers for Disease Control and Prevention’s Wide-Ranging Online Data for Epidemiologic Research (CDC WONDER) database [33]. Alcohol-related deaths were identified using the International Statistical Classification of Diseases and Related Health Problems, Tenth Revision (ICD-10), focusing on 14 alcohol-related causes of death. Each recorded death has a single underlying cause, with up to twenty additional contributing causes. Alcohol-related deaths are identified using specific codes from the International Statistical Classification of Diseases and Related Health Problems, Tenth Revision (ICD-10). These include K70 (alcoholic liver disease, ALD), F10 (mental and behavioral disorders due to the use of alcohol), and four codes for alcohol poisoning, R78, X45, X65, and Y15. ALD, mental and behavioral disorders due to the use of alcohol, and alcohol poisoning are the three main categories of alcohol-induced deaths. Other alcohol-related causes of death are listed under E24.4, G31.2, G62.1, G72.1, I42.6, K29.2, K85.2, and K86.

We analyzed mortality data for US individuals of all ages from 1999 to 2024. Death tallies from 1999 to 2023 are finalized, while data for 2024 remains provisional at the time of writing and may be subject to updates and changes. All provisional data were downloaded on January 28, 2025. Since the CDC WONDER database refers only to male and female deaths, we adopted a binary gender classification. The CDC WONDER database also provides state level data which includes the District of Columbia; thus, we examined data from all 51 jurisdictions referring to them as “states”. Finally, we analyzed data at the county level, of which there are 3,142 in 2024.

For data on mortality within 1999–2020, we followed the racial categorization provided by the CDC WONDER database, which uses “bridged race” categories listed as White, Black, American Indian and Alaska Native (AIAN), Asian and Pacific Islander (API). For mortality data from 2018 onwards “single race 6” categories, White, Black, AIAN, Asian, Native Hawaiian or other Pacific Islander (NHPI), and Mixed race, were used [33]. While both classifications are available for 2018–2020, “single race 6” is adopted exclusively from 2020 onwards. Thus, meaningful racial comparisons can be made only from 1999 to 2020 or from 2018 onwards. To derive fatalities for the Hispanic population, we selected “All races” and the origin subcategory “Hispanic or Latino.” To derive fatalities for all other races, we selected the race of interest and the origin subcategory “Not Hispanic or Latino.” Racial information is extracted from the death certificate of the deceased as reported by the funeral director, as provided by the next of kin, or on the basis of observation [33].

Race stratifications are based on “bridged race” categories for yearly data, and “single race 6” categories for monthly data. We utilized 10-year age groups, merging the 15-24 and 24-35 age populations for the monthly data due to small fatality numbers. For each demographic group, the yearly (monthly) crude rate is calculated as the number of fatalities in each year (month) divided by the associated total population in that given year (month), multiplied by 100,000. Population data were used to calculate crude rates, stratified by gender, age, race, and three cause-of-death categories: alcoholic liver disease (ALD), mental and behavioral disorders due to alcohol use, and alcohol poisoning. Yearly population estimates are made available by the Census Bureau and are included in the CDC WONDER database. Monthly population estimates are obtained from the online database on the US Census Bureau’s website [34,35]. When deriving the male-to-female ratio within a specific demographic, we divided the respective female and male crude rates. Relative changes in crude rates from year X to year Y are determined as a percentage, utilizing the formula, $100\left[\frac{CR(Y)}{CR(X)}-1\right]$, where CR(X) and CR(Y) are the crude rates for years X and Y, respectively. When displaying crude rates, we rounded them to one decimal place, but we evaluated their relative increases using their full numerical values. This may lead to small discrepancies between the stated crude rates and the resulting relative increases. All analyses were carried out using pandas, an open-source Python package included in the Anaconda distribution. Maps were created using the Python package geopandas.

### 2.3. Statistical analysis

We applied the Bayesian regression software Rbeast [36] to monthly crude rates between January 2018 and December 2024 for trend analysis. Rbeast can extract the trend and seasonal components of a (possibly noisy) time series and detect trend change points (TCPs) characterized by abrupt jumps between two consecutive trend curves across a single data point. Rbeast also provides 95% credible intervals for the trend segments and TCP locations. We utilized Rbeast to capture the trend in monthly crude rates, and to exclude seasonal effects and statistical fluctuations.

Mathematically, a time series can be approximated by a linear combination of a piecewise linear polynomial (trend function) and a piecewise trigonometric polynomial (seasonal function). Specifically, the trend function consists of linear segments joined at proposed trend change points (TCPs). The seasonal function is built by connecting trigonometric polynomials at seasonal change points (SCPs). Rbeast employs a Markov chain Monte Carlo (MCMC) algorithm to explore a wide range of possible models, which vary in the number and positions of TCPs and SCPs, the harmonic structure of the seasonal function, and the coefficients of the piecewise linear and trigonometric components. In this way, Rbeast estimates the posterior probability of the number and locations of TCPs and SCPs with uncertainty estimates. Also, Rbeast averages over all explored models to output the trend function $T\left(t_{i}\right)$ which describes the non-seasonal, deterministic component of the appropriate mortality at time point $t_{i}$. The trend function is comprised of a probability-weighted average of component trend functions, each describing a different statistical model with different sets of piecewise linear segments. The TCP is defined as $\begin{array}{c} \text{TCP}\left(t_{i}\right)≔T\left(t_{i}\right)-T\left(t_{i-1}\right) \end{array}$, the change in trend between its values at $t_{i}$ and $t_{i-1}$, and allows one to identify jumps or significant discontinuities in the trend. For a TCP to be classified as an abrupt jump within month $t_{i}$, we required that the relative TCP, $\frac{\text{TCP}\left(t_{i}\right)}{T\left(t_{i-1}\right)}$, exceeded 5%. This threshold was chosen as it optimized the trade-off between sensitivity to true change points and robustness to minor fluctuations. Lowering the threshold to 3% resulted in the detection of a few additional TCPs, while increasing it to 10% led to the exclusion of only a small number of reported TCPs. Most importantly, the most significant TCPs, occurring in Spring 2020 and concurrent with the COVID-19 pandemic, were retained across our sensitivity analyses of the percentage threshold. We used Rbeast with the default setting that allows the detection of up to 10 TCPs and SCPs, respectively. Since MCMC is a stochastic algorithm, repeated runs may yield slightly different regression results. To ensure Rbeast converged to the underlying probability distribution of the number and locations of TCPs, we configured the MCMC algorithm with 10 independent chains, each consisting of 100,000 samples [37,38]. This setting led to highly stable regression results so that the number and locations of the detected TCPs remained the same across different runs. Any differences emerged only in the credible intervals, with deviations of up to plus or minus one month and trend values (with deviations up to plus or minus 0.02). In our analyses, Rbeast consistently returned a seasonal component whose magnitude is much smaller compared to the trend component. Additionally, Rbeast returned zero SCPs in most cases and, at most, identified one SCP with insignificant jumps. As a result, throughout the rest of this paper, we omit discussions of SCPs.

## 3. Results

### 3.1. Nationwide

#### 3.1.1. Crude rates, 1999–2024 (age, gender) and 1999–2020 (race, gender).

**Fig 1:**
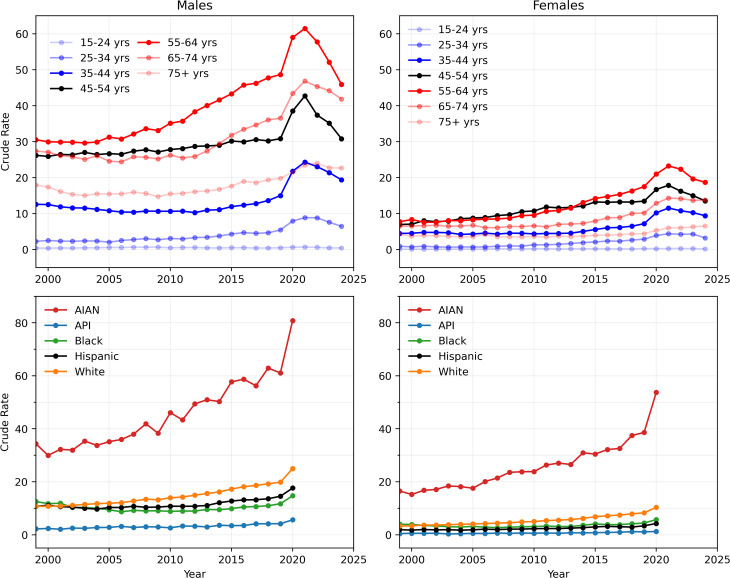
Yearly crude rates stratified by age from 1999 to 2024 and by race from 1999 to 2020. Male mortality crude rates are always higher than female crude rates. The most affected groups are (by age) those aged 55-64, and (by race) AIAN populations. Racial stratification is based on “bridged race” categories and cannot extend beyond 2020 since racial categorizations in the CDC WONDER database were changed that year. Yearly crude rates for a given demographic group are defined as the number of fatalities of that group in a given year, divided by the entire population, multiplied by 100,000.

We assessed the long-term dynamics of alcohol-induced deaths in the United States between 1999 and 2024. Overall, crude rates for alcohol-induced deaths of all ages increased by 89%, rising from 7.0 in 1999 to 13.2 in 2024. Annual fatality peaked in 2021 with 54,258 deaths. To obtain a more resolved picture, we evaluate age- and race-stratified yearly crude rates for both males and females, between 1999 and 2024 (age stratification) and between 1999 and 2020 (race stratification) and plot them in Fig 1. Race stratification was possible only until 2020 when the CDC WONDER classification criteria were changed.

Fig 1 shows that crude rates are always highest among males, for all ages and races. Among males, age stratification reveals that crude rates are highest among those aged 55–64 over the entire 1999–2024 period. Starting from the mid 2010s, the crude rate for females is also highest in this age cohort. In earlier years, crude rates in this age group were comparable to that of individuals aged 45–54. Notably, while male crude rates are roughly stable until 2019 for younger cohorts, they begin to gradually increase around 2010 for older groups, particularly those aged 55–64, and to a lesser degree, those aged 65–74. For example, while in 1999 crude rates did not differ much between males aged 55–64 and males aged 45–54 (30.5 and 26.2 fatalities per 100,000, respectively) in 2019, crude rates for the former group were about 60% larger than for the latter (48.7 and 30.8 fatalities per 100,000, respectively).

Race stratification indicates that, for both genders, the highest crude rates occur in the AIAN population. For males in this group, crude rates were almost three times that of males of all other races in 1999 (34.4 fatalities per 100,000 AIAN males compared to 12.5 fatalities per 100,000 Black males, the group with the second-highest crude rate in 1999) and more than three times that of males of other races in 2020 (80.8 fatalities per 100,000 AIAN males compared to 25 fatalities per 100,000 White males, the group with the second highest crude rate in 2020). Similarly, AIAN females exhibit crude rates that were more than four times higher than those of females of other races in 1999 (16.5 fatalities per 100,000 AIAN females compared to 4.0 fatalities per 100,000 Black females, the group with the second-highest crude rate in 1999), and more than five times higher in 2020 (53.8 fatalities per 100,000 AIAN females compared to 10.4 fatalities per 100,000 White females, the group with the second-highest crude rate in 2020).

#### 3.1.2. Relative crude rates, 1999–2024 (age, gender) and 1999–2020 (race, gender).

**Fig 2:**
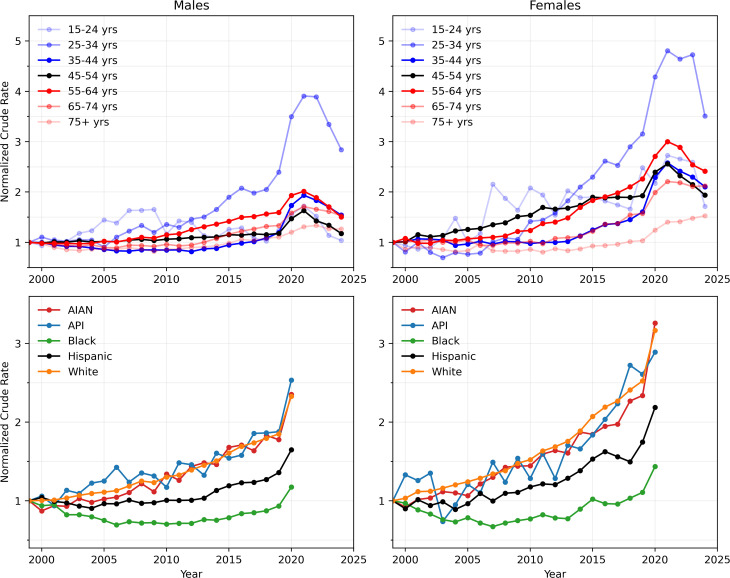
Yearly crude rates normalized by their 1999 value. Values below 1 indicate crude rate decreases relative to their 1999 value, those above 1 indicate increases. The largest crude rate increases relative to their 1999 values are among females for all ages and races. By age, the largest increases compared to 1999 are among those aged 25-34 for both genders; by race, the largest increases occurred in the AIAN, API, and White populations, for both genders. Non-normalized crude rates are shown in Fig 1.

To better visualize how alcohol-induced mortality changed over time, we plot crude rates normalized relative to their 1999 values in Fig 2. First, we note that although the overall number of female deaths were lower than that of males for all ages and races, as seen in Fig 1, crude rates among females increased at a much higher rate than those of males, for all ages and races. Age stratification reveals that the largest increases in mortality since 1999 occurred among those aged 25–34 and that, for all ages under 75, mortality peaked in 2021, at the height of the pandemic. Among males in this cohort, crude rates rose from 2.3 fatalities per 100,000 in 1999 to 8.9 fatalities per 100,000 in 2021 (a 291% increase from 1999), before decreasing to 6.5 fatalities per 100,000 in 2024 (a 188% increase from 1999). Among females, crude rates rose from 0.9 fatalities per 100,000 in 1999 to 4.4 fatalities per 100,000 in 2021 (a 381% increase from 1999) and have slightly decreased to 3.2 fatalities per 100,000 in 2024 (a 255% increase from 1999).

Fig 2 also shows that together with the API and White populations, the AIAN population experienced the largest increase in crude rates between 1999 and 2020, for both genders, with increases of 135% among males and 226% among females. In this same period, crude rates increased at a much slower rate among Hispanics. Remarkably, alcohol-induced mortality for Black males and females decreased until the mid-2010s and crude rates remained below their 1999 values until 2019, rising again in 2020. For all age and race groups, and relative to 1999 levels, crude rates have risen more sharply among females than males, pointing to the growing severity of alcohol-induced mortality among women.

#### 3.1.3. Male-to-female crude rate ratios, 1999–2024 (age) and 1999–2020 (race).

**Fig 3:**
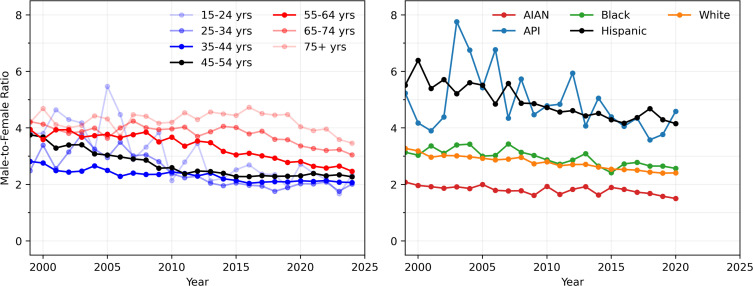
Male-to-female ratios of yearly crude rates. Although all ratios are consistently above unity, they are steadily decreasing across all age and race groups, particularly among younger cohorts. AIAN populations display the lowest male-to-female ratio. Gender disparities are largest among Hispanic and API populations where in 2020 the male crude rate was more than four times that of females. The crude rates of male and female alcohol deaths used to calculate these ratios are shown in Fig 1.

To provide an overview of possible disparities in alcohol-related deaths between the two genders, in Fig 3, we plot ratios of the male crude rate to the female crude rate from 1999 to 2024 for all ages and races. If we assume that male and female populations are approximately equal in number, the male-to-female crude rate ratio is approximately the same as the male-to-female death ratio. Overall, the male-to-female crude ratio has decreased steadily across most age groups and racial categories, underscoring that alcohol-induced mortality is becoming an increasingly significant problem among females. Younger age groups exhibit lower male-to-female ratios, with the gender gap decreasing over time. AIAN populations have a distinctly low male-to-female ratio, decreasing from 2.1 fatalities per 100,000 in 1999 to 1.5 fatalities per 100,000 in 2020. Male-to-female ratios in White and Black populations are similar at 3.3 and 3.1 fatalities per 100,000, respectively, in 1999, and at 2.4 and 2.6 fatalities per 100,000, respectively, in 2020. API and Hispanic populations exhibited the largest male-to-female ratios, at around 5.2 and 5.5 fatalities per 100,000 in 1999, respectively, and 4.6 and 4.2 fatalities per 100,000 in 2020, respectively.

#### 3.1.4. Crude rates, 2018–2024 monthly trends (age, gender, cause of death).

**Fig 4:**
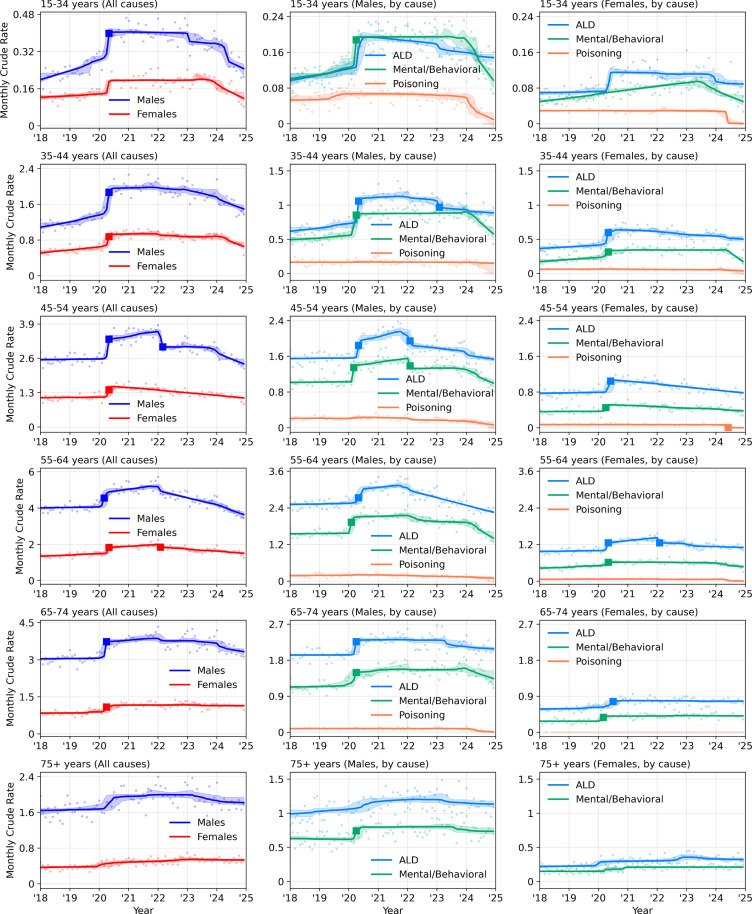
Alcohol-related monthly crude rates between 2018 and 2024, stratified by gender, age and cause of death. Data are indicated as dots, solid curves represent Rbeast-derived trends, shaded areas their CIs, solid squares indicate statistically significant TCP jumps. The first column displays results for all causes of death stratified by gender and age, the second and third columns display results further stratified by cause of death. Except for those aged 75 and over, all ages experienced a statistically significant TCP jump in the Spring of 2020. Males and females aged 55-64 display the largest mortality, but the largest relative increase was among males aged 15-34 (+28%, between April and May 2020) and females aged 35-44 (+28%, between April and May 2020). TCP jump values are listed in Table A in S1 Text for males, and Table B in S1 Text for females.

The previous sections reveal steep upward trends in alcohol-induced deaths starting in 2020, for all demographic groups. Nationwide, alcohol-induced deaths were 39,043 in 2019 (11.9 fatalities per 100,000), and 54,258 in 2021 (16.3 fatalities per 100,000), corresponding to a 39% increase between 2019 and 2021 (and a 37% increase in the crude rate). In 2024, there were 44,168 alcohol-induced deaths in the country (13.2 fatalities per 100,000) corresponding to a 13% increase compared to 2019 (and an 11% increase in the crude rate). The increase in crude rate between 2019 and 2021 was slightly higher among females than males (+39% for females, + 36% for males); and similarly, between 2019 and 2024 (+16% for females, + 8% for males). These figures imply that, nationwide, mortality increased significantly at the height of the COVID-19 pandemic, and that while crude rates have begun decreasing, in 2024 they are still larger than they were in 2019. Building on reports of accelerated alcohol-related deaths during the COVID-19 pandemic [23,25,39], we performed a detailed monthly trend analysis (rather than a yearly analysis) to gain better insight of the impact of alcohol-induced mortality across demographic groups between 2018 and 2024. We also wish to determine for which groups, if any, mortality rates have decreased or returned to pre-pandemic levels. Specifically, we apply Rbeast to monthly crude rates to identify possible upward TCP jumps in mortality trends. We find that statistically significant TCP jumps arise for both genders and all age groups below 75 years in Spring 2020, concurrent with the onset of COVID-19. As shown in Fig 4, the largest TCP jumps occurred in younger cohorts, with male mortality trends increasing by 28% for ages 15–34 between April and May 2020, and by 26% for ages 35–44 in the same period. Among females, the largest TCP jump was for those aged 35–44 whose mortality trend rose by 28% between April and May 2020. For both genders and all age groups less than 75 years, trends remained abnormally large throughout 2023, despite some groups experiencing downward TCP jumps. For most groups, significant decreases start emerging only in 2024, suggesting long-lasting effects of approximately 4 years.

Further stratification of monthly crude rates by cause of alcohol-induced death for both genders are also shown in Fig 4. ALD emerges as the main cause of death, followed by mental and behavioral disorders due to alcohol use, especially for younger cohorts. Alcohol poisoning remains uniformly low and is not associated to any TCP jump. For males aged 75 and over, and for females aged 65 and over, mortality cases due to alcohol poisoning were extremely rare and suppressed in most months between 2018 and 2024. Consequently, the application of Rbeast for trend analysis is not feasible for older populations due to insufficient data. Among females, the upward TCP jumps for ALD are of the same magnitude as for all alcohol-induced deaths, implying that the mortality surge observed in Spring 2020 is mostly attributable to ALD rather than to mental or behavioral disorders. Conversely, males aged 35–44 have a TCP jump in overall mortality in May 2020 (change: 0.39) that can be attributed to TCP jumps both in ALD (May 2020, change: 0.31) and in mental and behavioral disorders (April 2020, change: 0.15). Stratification by cause of death reveal that mortality due to alcohol-induced mental and behavioral disorders increased by 43% among males aged 15–34 in the month between March and April 2020. ALD deaths rose by 42% for males aged 35–44, and by 35% for females aged 35–44, both in the month between April and May 2020. Fig 4 also shows that crude rates for alcohol-induced deaths due to mental and behavioral remain elevated through 2023 for most age cohorts. Crude rates begin decreasing in later months and by the end of 2024 they are comparable to, and in some cases less than, their 2019 counterparts. This is especially true for cohorts younger than 65 years. However, ALD crude rates for cohorts younger than 65 remain in excess of their pre-pandemic values throughout 2024.

#### 3.1.5. Crude rates, 2018–2024 monthly trends (race, gender).

**Fig 5:**
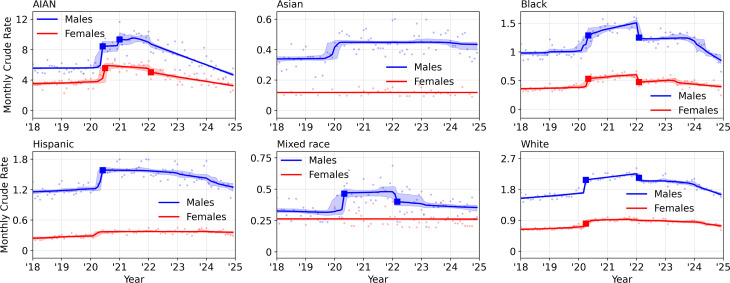
Alcohol-related monthly crude rates for ages 15 and older between 2018 and 2024, stratified by gender and race. Males of all races, except Asian, experience a TCP jump in early 2020. The TCP jumps are: AIAN: + 41% males (May to June 2020) and +32% females (June to July 2020); Black: + 14% males (April to May 2020) and +32% females (April to May 2020); Hispanic: + 15% males (May to June 2020); Mixed race +37% males (April to May 2020); White: + 22% males (March to April 2020) and +12% females (March to April 2020). Mortality trends are much higher among males than females. Data are indicated by dots, solid curves represent Rbeast-derived trends, shaded areas represent their CIs, and solid squares mark statistically significant TCP jumps. Racial stratification is based on the “single race 6” classification. TCP jump values and their CI are listed in Table C in S1 Text.

We now stratify alcohol-induced mortalities between 2018 and 2024 using all racial categories listed under “single race 6,” and including all ages. Results are shown in Fig 5 and reveal distinct racial patterns. Males of all races experience significant upward TCP jumps during the of Spring 2020, concurrent with the onset of the COVID-19 pandemic, except for Asian males. AIAN males suffered from disproportionately high alcohol-induced mortality compared to other races even prior to the pandemic, as discussed earlier. Here, we note that their pre-TCP monthly crude-rate trend value (6.0 fatalities per 100,000 in May 2020) is more than three times higher than the next largest one (1.7 fatalities per 100,000 in Mar 2020) for White males. AIAN males also experienced the highest TCP jump during the Spring of 2020, with a post-TCP trend value (8.4 fatalities per 100,000 in June 2020), that corresponds to a 41% rise in one month, an increase that is much higher than that of other races. A subsequent upward TCP jump (9.3 fatalities per 100,000 in January 2021) increased the alcohol-induced mortality trend ever further. AIAN females had the highest pre-TCP monthly crude-rate trend value (4.2 fatalities per 100,000) in June 2020. They also experienced the largest relative increase, rising by 32% from June to July 2020. This increase was on par with Black females, whose mortality trend also increased by 32% between April and May 2020. For comparison, the TCP jump among Black males is 14% between April and May 2020.

### 3.2. States

#### 3.2.1. Crude rates, yearly data 1999–2024.

The above analyses were conducted using data aggregated at the national level. In this section we study how alcohol-induced deaths vary by state within the entire 1999–2024 period but with a specific focus on 2019–2024. Due to the sparsity of the data, it is not possible to conduct concurrent stratification of alcohol-induced deaths by age, gender, and race in each state, especially in the pre-pandemic year 2019. It is also not possible to perform Rbeast analyses on monthly data for individual states. We thus pool mortality across all races and ages to determine how crude rates for males and females varied in each state between 1999 and 2024.

For all states, crude rates have risen in the 26-year period under investigation. The state with the largest averaged crude rate is New Mexico, followed by Alaska and Wyoming. Starting in 2010, New Mexico has also consistently been the state with the largest crude rate. Interestingly, in the early 2000s the District of Columbia was among the regions with the largest crude rates; however, in more recent years its values have been surpassed by those of several other states. Hawaii is the state with the lowest overall crude rates between 1999 (3.1 fatalities per 100,000) and 2024 (6.3 fatalities per 100,000) followed by Maryland and Pennsylvania. The highest crude rates were recorded during the pandemic year 2021, specifically in New Mexico (51.6 fatalities per 100,000), Alaska (43.0 fatalities per 100,000) and South Dakota (42.1 fatalities per 100,000). All states had record crude rates in the pandemic years 2020, 2021 or 2022, compared to other years. In Nebraska, Kentucky, Connecticut, Massachusetts, New Jersey crude rates were at their highest in 2020. In Oregon, New Hampshire, Minnesota, Wisconsin, Delaware, Indiana, Illinois, New York, crude rates were highest in 2022. In all other states, crude rates peaked in 2021.

To better understand how alcohol-related mortality patterns evolved during and after the COVID-19 pandemic, we now compare the 2019 crude rates in each of the 50 states plus the District of Columbia to their 2021 and 2024 counterparts. These comparisons help us identify the geographic areas in which crude rates rose the most and the least in 2021, at the height of the pandemic, compared to 2019, just before the pandemic. Statewide analyses will also reveal the regions where, by 2024, crude rates had returned to levels comparable to those in 2019.

Between 2019 and 2021, crude rates rose significantly in all states. The largest increases occurred in Mississippi, where crude rates rose from 8.1 to 17.9 deaths per 100,000 (+122%), by South Dakota (+95%) and Alaska (+70%). The state with the smallest 2019–2021 increase is Vermont (+12%), followed by Arkansas (+20%) and Oklahoma (+26%). Among the five states for which crude rates peaked in 2020, the more contained increases between 2019 and 2020 were in New Jersey (+23%), followed by Kentucky (+33%). Among the eight states for which crude rates peaked in 2022, the smallest increase with respect to 2019 values occurred in New York (+25%) followed by Oregon and Wisconsin (+34% for both).

A similar analysis shows that by 2023 crude rates were still at least 10% larger than compared to their 2019 values in all states, except for Wyoming, New Jersey, Hawaii and Vermont whose crude rates were slightly below their 2019 levels. However, by 2024 crude rates decreased substantially and remained 10% above their 2019 values in about only half of all states. The largest decrease occurred in New Jersey where 2024 crude rates are 20% below their 2019 values, followed by Hawaii and Oklahoma, whose 2024 crude rates are both 6% below their 2019 values. Among jurisdictions where 2024 crude rates remain above their 2019 values, the state with the largest imbalance remains Mississippi, where in 2024 the crude rate was still 70% larger compared to its 2019 level. Mississippi is followed by South Dakota and Iowa for which crude rates are 54% and 30%, respectively, larger compared to their 2019 values. Interestingly, in New Mexico, the state with the largest crude rate in all years since 2010, crude rates in 2024 (33.8 fatalities per 100,000) returned to values similar to the ones recorded in 2019 (34.8 fatalities per 100,000).

#### 3.2.2. Crude rates, yearly data 1999–2024 (gender).

Upon stratifying by gender, we observe that between 1999 and 2024 the largest increases in crude rates for males were recorded in Idaho (+210%), South Dakota (+202%), and North Dakota (+190%). While crude rates among males increased in all other states, in the District of Columbia they decreased by 32%. This is the only jurisdiction were crude rates fell within the 26-year period under investigation. Among females, states with the largest relative increases between 1999 and 2024 are Iowa (+365%), Oregon (+349%) and Minnesota (+289%). Similarly, between 1999 and 2024 crude rates decreased by 26% in the District of Columbia. There were not sufficient female deaths in 1999 to determine crude rates for Delaware, Hawaii, Montana, North Dakota, Rhode Island, Vermont, and Wyoming.

**Fig 6:**
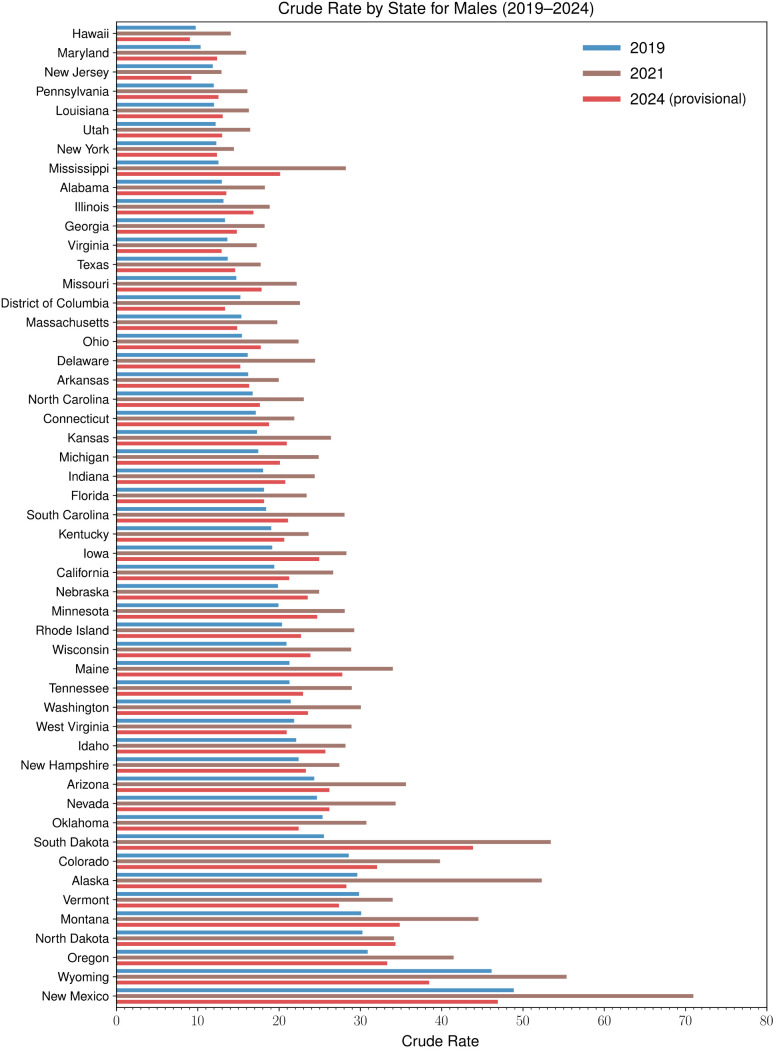
Yearly crude rates for alcohol-induced deaths among males by state in 2019, 2021, and 2024. In 2019, states with the largest crude rates among males were New Mexico (48.9 fatalities per 100,000), Wyoming (46.1 fatalities per 100,000), and Oregon (30.9 fatalities per 100,000). In 2021, at the height of the pandemic, the largest crude rates among males were recorded in New Mexico (71.0 fatalities per 100,000), Wyoming (55.4 fatalities per 100,000), and South Dakota (53.4 fatalities per 100,000). By 2024, crude rates had decreased with respect to their 2021 values in all states except in North Dakota where crude rates further increased from 34.1 to 34.3 fatalities per 100,000. In 2024, crude rates among males were largest in New Mexico (46.9 fatalities per 100,000), South Dakota (43.9 fatalities per 100,000), and Wyoming (38.5 fatalities per 100,000). In 21 states, crude rates remained above their 2019 values by at least 10%. The ones with the largest relative rises are South Dakota (+72%), Mississippi (+60%), and Maine (+31%). Conversely, in 2024 crude rates among males had decreased compared to their 2019 values in 12 states. The largest declines between 2019 and 2024 were recorded in New Jersey (-22%), Wyoming (-17%), Oklahoma (-12%), and the District of Columbia (-12%). These results reveal that while in some states the mortality surge observed in 2021 has mostly subsided, in others, long-lasting effects persist.

**Fig 7:**
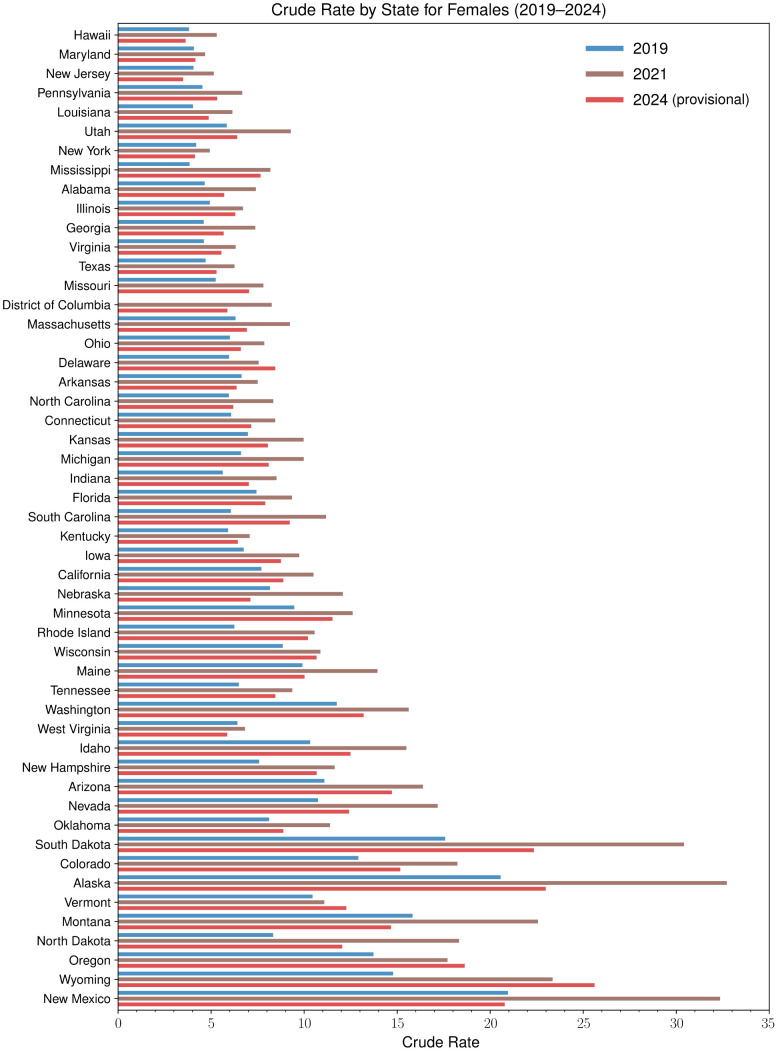
Yearly crude rates for alcohol-induced deaths among females by state in 2019, 2021, and 2024. In all states and years, crude rates among females are lower than among males. In 2019, New Mexico (21.0 fatalities per 100,000), Alaska (20.6 fatalities per 100,000), and South Dakota (17.6 fatalities per 100,000) recorded the largest crude rates for female fatalities. In 2021, at the height of the pandemic, crude rates increased in all states compared to 2019; they were greatest in Alaska (32.7 fatalities per 100,000), New Mexico (32.4 fatalities per 100,000), and South Dakota (30.4 fatalities per 100,000). By 2024, crude rates still exceeded their 2019 values by at least 10% in 33 states. Among females, 2024 crude rates were largest in Wyoming (25.6 fatalities per 100,000), Alaska (23.0 fatalities per 100,000), and South Dakota (22.3 fatalities per 100,000). The largest relative increases between 2019 and 2024 crude rates occurred in Mississippi (+99%), Wyoming (+73%), and Rhode Island (+63%). Crude rates decreased the most between 2019 and 2024 in New Jersey (-14%), Nebraska (-13%), and West Virginia (-9%). These data reveal that the 2021 rises in mortality have been longer-lasting among females than males.

To better investigate how mortality trends changed after the advent of COVID-19 we show crude rates as recorded in 2019, 2021 and 2024 for males and females in each state in Figs 6 and 7. As can be seen, between 2019 and 2021 crude rates increased significantly for both genders in all jurisdictions for which data is available. Although New Mexico is the state with highest male crude rate in all three years, the largest relative increases were recorded between 2019 and 2021 in Mississippi (+125%), South Dakota (+109%), and Alaska (+77%). Among females, New Mexico is the state with the highest crude rate until 2019, surpassed by Alaska between 2020 and 2023, and finally by Wyoming in 2024. The largest relative increases for females since 2019 occurred in Mississippi between 2019 and 2022 (+151%), North Dakota between 2019 and 2021 (+120%), and Rhode Island (+107%) between 2019 and 2022.

**Fig 8:**
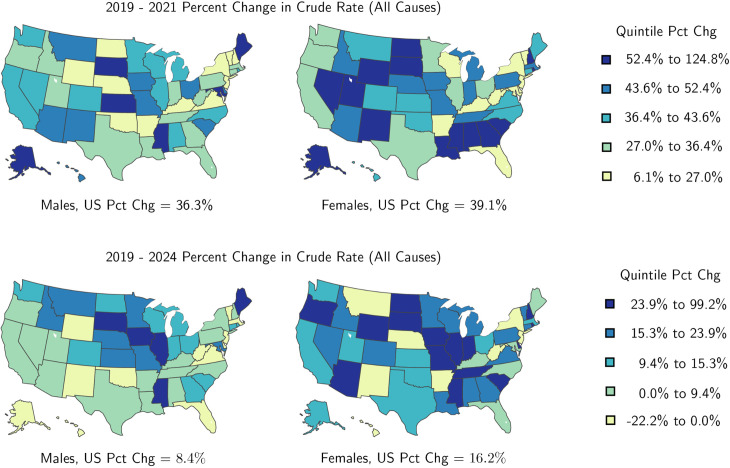
Percent change in yearly crude rates (between 2019 and 2021 and between 2019 and 2024) by gender and state. Between 2019 and 2021 alcohol-induced mortality increased in all states, for both genders. In Mississippi the overall crude rate rose by +122% in two years (+125% among males and +113% among females). The relative rise in female crude rates exceeds, or is roughly on par with, that of males in most states, revealing fast-paced increases in alcohol-induced mortality among females. In 2024, crude rates were still elevated compared to the respective 2019 values, but to a lesser degree. In Mississippi, the 2024 overall crude rate was still 70% larger than in 2019 (+60% among males, and +99% among females). The percent change values listed under each map are obtained by tallying alcohol-related deaths in the entire country. Compared to 2019, 2024 crude rates were still significantly higher among females (16.2%) than among males (8.4%). All crude rates and percentages are listed in Table D for males and in Table E in S1 Text for females.

While crude rates are always higher among males, in 26 out of the 50 states for which data is available, the corresponding relative increases from 2019 to 2021 among females surpassed those among males. Of these 26 states, in 19 of them the differential exceeds 10%. The largest gender gap is in North Dakota, where female crude rates increased by 120%, and male crude by 13% between 2019 and 2021, followed by Wyoming, where female crude rates rose by 58% and male crude rates by 20% in the same period. These trends are reversed in the other 24 states. Among them, the largest gender imbalances are observed in Maryland where male crude rates rose by 54% while female crude rates rose by 15%, followed by South Dakota where male crude rates rose by 109% while female crude rates rose by 73%. Corresponding rates are listed in Table D in S1 Text for males, and in Table E in S1 Text for females. In Fig 8, we show relative percent changes in each state between 2019 and 2021, and between 2019 and 2024, and report on the corresponding percent change within the entire country. Whereas the 2019–2021 relative increases for each gender are comparable, by 2024 crude rates were still significantly higher than in 2019 among females (16.2%) than among males (8.4%).

#### 3.2.3. Crude rates, 2019–2024 yearly trends (cause of death, gender).

We now stratify alcohol-induced deaths by gender and cause of death in each state. The three main causes of death are ALD, deaths from mental or behavioral issues due to alcohol, and alcohol poisoning.

Between 2019 and 2021, ALD deaths among males increased in all states. Relative increases in male crude rates were particularly elevated in Mississippi (+164%), South Dakota (+129%) and Alaska (+115%). Crude rates increased the most among females in Mississippi as well (+182%), followed by Utah (+104%) and South Carolina (+95%). Interestingly, between 2019 and 2021 in West Virginia ALD crude rates decreased by 4% among females. In 30 states, the relative increase for ALD crude rates between 2019 and 2021 was higher for females than males. Due to low death counts (less than 10), female crude rates from ALD mortality are unavailable in Delaware, Hawaii, and Rhode Island in 2019 and in the District of Columbia for both 2019 and 2021.

After 2021, ALD crude rates subsided for both genders, without returning, for the most part, to pre-pandemic levels. Compared to their peak levels, ALD crude rates decreased more among males than females, so that in most states the relative rises between 2019 and 2024 remain higher among females. Mississippi still displays the largest relative increase between 2019 and 2024, for both genders (+98% for males and +159% for females). By 2024, male crude rates for ALD-related deaths had fallen below 2019 levels in eleven states. In contrast, only five states experienced a decline in female crude rates over the same period. Out of the 35 states where 2024 ALD crude rates remained above their 2019 values for both genders, relative increases among females were higher than among males in 26 of them. These findings indicate that females were generally more vulnerable to ALD mortality compared to males, and that ALD was a major determinant in the rising levels of alcohol-induced female mortality.

Relative increases from 2019 to 2021 in crude rates for fatalities due to alcohol-induced mental and behavioral disorders were particularly elevated in South Dakota (+101%), South Carolina (+77%), and Washington, Michigan, Missouri (+74% for these three states) among males, and Tennessee (+94%), Georgia (+82%), Alaska (+78%) among females. While crude rates among males rose in all states from 2019 to 2021, there were two states for which they decreased among females, Maryland (-7%) and New Jersey (-5%). There were not enough female fatalities due to alcohol-induced mental and behavioral disorders in Delaware, the District of Columbia, Hawaii, North Dakota, Rhode Island, Vermont, and Wyoming in 2019 for meaningful comparisons.

By 2024, crude rates for fatalities due to alcohol-induced mental and behavioral disorders had decreased compared to their 2021 levels in most states and for both genders. In general, these decreases were more modest compared to the decreases observed for mortality due to ALD, suggesting longer lasting impacts of alcohol-induced mental and behavioral disorders. Among males, 2024 crude rates remained higher than in 2019 in 38 states; among females, 2024 crude rates exceeded their 2019 values in 34 states. The relative differences between 2024 and 2019 crude rates are highest in South Dakota (+107%), Nebraska (+77%) and Maine (+68%) for males, and in South Carolina (+66%), Tennessee (+57%) and Arizona (+54%) for females. Between 2024 and 2019, crude rates declined in 13 states for males and in 9 states for females. Crude rates due to alcohol-induced mental and behavioral disorders cannot be determined in 2024 for females in Delaware, the District of Columbia, Hawaii, Rhode Island, Vermont, and West Virginia due to low fatalities.

No clear patterns arise for changes in crude rates due to alcohol-induced poisonings between 2019 and 2021, either because of relatively few fatalities or because, in some states, crude rates rose, and in others, they fell. For states where crude rates can be evaluated, however, it is noteworthy that by 2024, crude rates for both genders had decreased in all states compared to their 2019 levels.

The relative changes in crude rates for mortality due to ALD, mental and behavioral disorders from alcohol use, and alcohol-induced poisoning, respectively, between 2019 and 2021, and between 2019 and 2024, for both genders and in all states are shown in Fig A in S1 Text, Fig B in S1 Text, and Fig C in S1 Text, respectively. For ALD deaths nationwide, 2024 crude rates were significantly higher than in 2019 among females (17.6%) than among males (6.5%). These findings support the notion that ALD mortality has impacted females more than males; conversely, mental and behavioral disorders from alcohol use impacted both genders, in roughly equivalent manner.

### 3.3. Counties

#### 3.3.1. Crude rates, yearly data 1999–2024.

Finally, we perform a county-level analysis for a more local view on how alcohol-induced deaths affected various communities. The number of counties in the United States has changed over the 1999–2024 time-frame due to the merging of existing counties and the creation of new ones. In 2024, the number of counties in the United States is 3,142, including the District of Columbia. The CDC WONDER database allows for meaningful evaluation of crude rates only in about 1,300 of them depending on the year; the number fluctuates due to changes in mortality since the CDC does not report fatality counts when they are less than 10. Of the counties for which enough data exists, the ones most impacted by alcohol-induced mortality over the 26-year period between 1999 and 2024 are Oglala Lakota County (SD) (known as Shannon County until 2015), McKinley County (NM), Apache County (AZ), Rio Arriba County (NM), and Navajo County (AZ).

**Fig 9:**
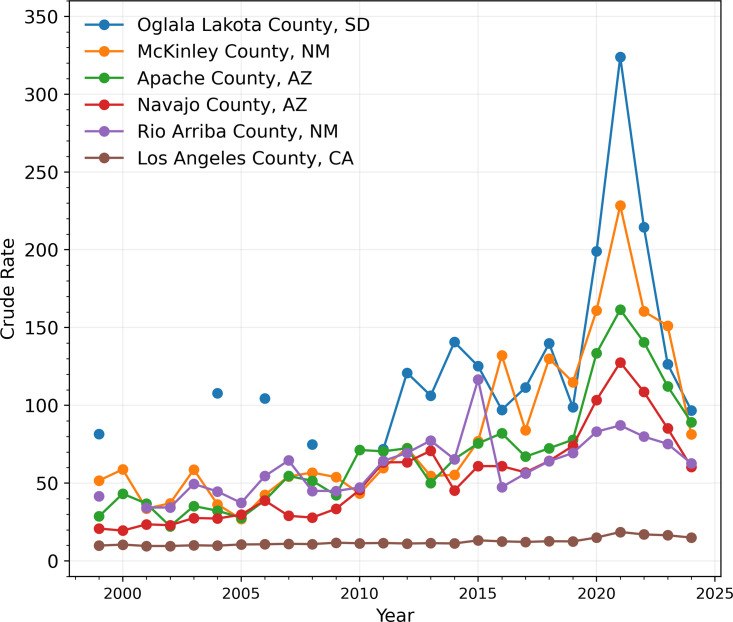
Crude rates in select US counties between 1999 and 2024. The counties chosen are the five with the highest average crude rate over the 26-period between 1999 and 2024, and Los Angeles County, the most populous in the nation and the one with the highest number of fatalities. Los Angeles County is included for comparison. Prior to 2011, Oglala Lakota County had too few fatalities for a meaningful evaluation of the crude rate. In 2021, the number of residents in each of the counties shown was approximately 13,600 in Oglala Lakota County (SD), 72,000 in McKinley County (NM), 65,600 in Apache County (AZ), 108,000 in Navajo County (AZ), 40,200 in Rio Arriba County (NM) and 9,800,000 in Los Angeles County (CA). Crude rates reached record high values in 2021 for all counties shown; by 2024 they had returned to values comparable to 2019 levels.

These counties report the highest crude rate averages between 1999 and 2024, at over 50 fatalities per 100,000. In particular, Oglala Lakota County (SD), McKinley County (NM), and Apache County (AZ) have reported staggering alcohol-induced crude rates of over 80 fatalities per 100,000 every year since 2020. In Fig 9, we show longitudinal trends (1999–2024) of the crude rates in these five counties; we also include values from Los Angeles County, the most populous in the country and the one with the highest number of alcohol-induced fatalities, for reference.

**Fig 10:**
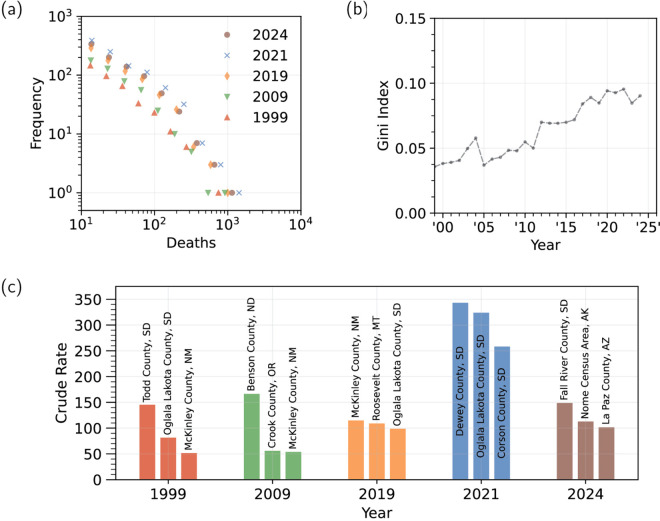
Alcohol-induced fatalities and crude rates in the United States at the county level. (a) Histogram of alcohol-induced fatalities for select years. Fatalities were highest in 2021. (b) Gini index evaluated for all years between 1999 and 2024. A Gini index of 0 implies that the crude rate is the same for all counties. If all alcohol-induced fatalities occurred in one out of N_c_ counties, the Gini index would be 1 − 1/N_c_. The number of counties with statistically significant fatality counts (>10 deaths in a given year) and crude rates are N_c _= 1,345 in 1999 and N_c_ = 1,303 in 2024. Although slightly increasing, the Gini index remains below 0.1 for all years, suggesting a relatively uniform incidence across counties irrespective of their population. (c) The three counties with the largest crude rates in 1999, 2009, 2019, 2021 and 2024.

In Fig 10a, we show the frequency distribution of counties that recorded more than 10 alcohol-induced deaths for select years between 1999 and 2024. The number of counties that reached this level increased from 1,375 (out of 3,147) in 1999 to 1,380 (out of 3,142) in 2021, as determined from the CDC WONDER database. Los Angeles County, the most populous in the United States, is consistently the one with the most deaths. The number of counties reporting more than 100 deaths increased steadily between 1999 (24 counties) and 2019 (66 counties), and peaked in 2021 (108 counties). The tally for 2024 (78 counties) is less than the 2021 peak, and is comparable to what recorded in 2021. In 1999, only Los Angeles County had more than 400 yearly alcohol-induced deaths (930 fatalities); by 2019 this was also true of Maricopa County (AZ), San Diego County (CA) and Cook County (IL). In 2021, 10 counties had more than 400 yearly deaths, with Los Angeles County (1828 fatalities) doubling its 1999 count. By 2024, values had decreased in all of them, with only six reaching 400 deaths, and with Los Angeles County recording the most (1450 fatalities). The population in Los Angeles County was relatively stable at 9,437,290 in 1999; 9,829,544 in 2021 and 9,663,345 in 2024. In Fig 10c, we show the three counties with the highest crude rates in 1999, 2009, 2019, the pandemic year 2021, and 2024, the last year for which (provisional) data is available. Crude rates are highest during the pandemic year 2021. By 2024, the largest crude rates recorded were significantly lower than in 2021, but were still larger than in 2019.

**Fig 11:**
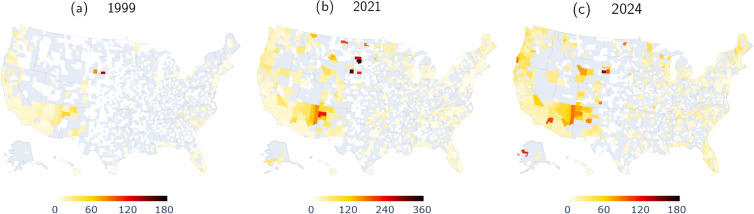
Maps of alcohol-induced crude rates in the United States at the county level for 1999, 2021, 2024. The scale of the color bar in 2021 differs by a factor of two compared to 1999 and 2024. Record crude rates were recorded in 2021. Comparisons between 2024 and 1999 reveal that crude rates rose significantly in the 26 years under investigation, especially in the South West, West and North East. In the gray counties, there were not enough fatalities for meaningful evaluation of crude rates.

To gain better insight on geographic variations, in Fig 11 we map crude rates for alcohol-induced fatalities for the entire country and for select years. Fig 11a shows that in 1999 the counties with the highest crude rates were in the Southwestern and Western regions of the United States, particularly Todd County (SD) where the crude rate was 145 fatalities per 100,000 and Oglala Dakota County (SD) where the crude rate was 82 fatalities per 100,000. In total, five counties had a crude rate larger than 30 fatalities per 100,000 in 1999. Over the next 20 years, crude rates increased throughout the country. In 2019 the county with the highest crude rate was recorded in McKinley County (NM) with 115 fatalities per 100,000 and Roosevelt County (MT) with 109 fatalities per 100,000. By 2019, the number of counties with a crude rate larger than 30 fatalities per 100,000 had increased to 61.

Crude rates surged dramatically in the pandemic year 2021 for all counties for which data is available, as can be seen in Fig 11b. Note that the scales of the color bars in Figs 11a and 11b differ by a factor of two. For most counties, crude rates peaked in 2021, for a few others, record highs were recorded either in 2020 or 2022. By 2021, crude rates had surpassed 200 fatalities per 100,000 in six counties: Dewey County (SD), Oglala Lakota County (SD), Corson County, (SD) McKinley County (NM), Todd County (SD), Roosevelt County (MT), in decreasing order. The largest value was in Dewey County (SD) which recorded a crude rate of 343 fatalities per 100,000. In 2021, 173 counties reported in excess of 30 fatalities per 100,000.

By 2024, crude rates had decreased from their respective peaks in all counties, and were larger than 100 fatalities per 100,000 only in three counties, and larger than 30 fatalities per 100,000 in 87 of them. Fig 11c shows 2024 crude rates using the same color bar scale as in 1999. Upon comparing Figs 11a and 11c, it is clear that between 1999 and 2024, the incidence of alcohol-induced mortality has risen for all states for which data is available, although 2024 mortality is tempered compared to 2021.

These trends are confirmed by following the longitudinal evolution of the mean crude rates between 1999 and 2024. In 1999, the mean crude rate calculated for all counties for which data is available was 2.8 fatalities per 100,000; in 2009 it had risen to 4.2 fatalities per 100,000 and in 2019 it was 9.7 fatalities per 100,000. At the height of the pandemic, it reached 17.2 fatalities per 100,000 and in 2024 it had returned to 12.1 fatalities per 100,000.

Finally, to study how alcohol-induced mortality affects counties with different populations, in Fig 10b we plot the Gini coefficient [40] from 1999 to 2024. The Gini coefficient measures inequality among the values of a distribution, in our case, among the number of fatalities from the *N*_c_ counties that reported statistically significant numbers of alcohol-induced deaths. Specifically, the Gini index is determined as the proportion of the total number of alcohol-induced fatalities across all counties, against the cumulative population fraction across the same counties. The lower bound for the Gini index is 0 (perfect equality, indicating that alcohol-induced deaths and county populations are proportional), and the upper bound is 1 − 1/*N*_c_ (perfect inequality, indicating that all alcohol-induced deaths occurred within a single county). We find that, although steadily increasing, the Gini coefficient remains less than 0.1 in all years, including in the pandemic year 2021, indicating that county population is not a significant factor in alcohol-induced mortality, and that large and small population counties are affected in a proportional manner. Gini indices of less than 0.1 for alcohol-induced mortality align with comparable values observed for drug-overdose mortality in recent years [41]. However, while for alcohol-induced mortality the Gini index has not changed appreciably between 1999 and 2024, for drug-overdoses, it has decreased significantly from the peak values recorded in the early 2000s, which were in excess of 0.2. This indicates, that while drug-overdose deaths were initially concentrated in higher population centers and began affecting smaller counties in recent years, alcohol-induced mortality has impacted all counties for which data is available since 1999.

## 4. Discussion and conclusions

We analyzed the steady rise of yearly crude rates for alcohol-related fatalities between 1999 and 2024 and the abrupt rises that occurred starting in 2020 for all demographics. Yearly trends between 1999 and 2024 reveal that AIAN males have been the most affected group. Although mortality is highest among men for all demographic groups, crude rates are increasing faster among females, across all ages and races. Similarly, although mortality is highest among those aged 55–64 for both genders, the largest increases were for those aged 25–34. For females in this age group, crude rates increased almost four-fold between 1999 and 2024; for males they increased almost three-fold.

The use of Rbeast allowed us to identify changes in trends in the monthly crude mortality rates within age, race, gender groups, and causes of death. For all demographic groups, the month over which crude mortality rates jumped were between March and June 2020, near the start of COVID-19 pandemic. Upon stratifying by race and gender, we find that the most affected were AIAN males whose monthly crude rates rose by 41% in one month, and AIAN and Black females whose crude rates both rose by 32% in one month. Upon stratifying by age and gender, males aged 15–34 and females aged 35–44 were the most impacted groups; crude rates rose by 28% in one month for both groups. These abrupt rises are primarily linked to ALD (especially for females) and mental and behavioral disorders for both genders, with no notable increase in alcohol poisoning for either gender. A worthwhile direction for future work would be to examine whether, and to what degree, social isolation and disruptions in AUD treatment imparted by COVID-19 may have contributed to the large spikes in monthly crude rates observed in the Spring of 2020.

While most mortality trends began decreasing or stabilizing in 2023, only in 2024 did significant decreases emerge, and only for some demographic groups. This suggests that the COVID pandemic imparted effects that lasted at least 4 years. Crude rates for 2024 remain elevated compared to pre-pandemic levels especially among females with ALD. Furthermore, they are above their 2019 values by at least 30% in South Dakota, Mississippi and Maine among males, and in Mississippi, Wyoming and Rhode Island among females, where they exceeded their 2019 values by more than 60%. County-wise the highest crude rates were recorded in 2021, and occurred in areas with large AIAN populations in South Dakota and New Mexico. It would also be of great interest to investigate the impact of the COVID-19 pandemic on alcohol-related fatalities in countries other than the United States to facilitate cross-national comparisons and to inform global public health strategies [39,42,43].

Our findings extend the growing literature on increasing alcohol-induced mortality in the United States. Steady upward trends across gender and age groups have been reported for several decades [18–20], along with a narrowing gender gap [12,18,44]. More recent studies further describe sharp increases in mortality concurrent with the onset of the COVID-19 pandemic [23,25]. Our monthly data stratifications allowed us to unambiguously quantify these increases for all relevant demographic groups and to identify the statistically significant change points in the corresponding mortality trends. All occurred in Spring 2020, close to the start of the pandemic. Consistent with previous findings, we verified that these surges were driven primarily by deaths due to alcohol-related liver disease and alcohol-related mental and behavioral disorders [2,4,45,46]. What distinguishes our work from other studies is the examination of mortality trends beyond the acute phase of the COVID-19 pandemic. Following the time evolution of crude rates over an extended time horizon allowed us to identify the demographic groups for which mortality trends decreased the most, or in the most abrupt manner, compared to their peak values. Nevertheless, since as of 2024 alcohol-induced mortality remains elevated across all groups relative to pre-pandemic levels, continued surveillance and future analyses are needed to determine which demographic groups, if any, return to pre-pandemic baselines. Finally, whereas prior state-level research highlighted New Mexico and Alaska as long-standing outliers [21], our results reveal excess mortality in every state, with 2024 crude rates exceeding pre-pandemic levels by at least 10% in roughly half of them. This work provides a longer post-pandemic time-series analysis compared to the existing literature and offers a detailed demographic and geographic view of alcohol-induced deaths.

### 4.1. Limitations

The underlying causes of death are selected from a list of alcohol-induced causes, as itemized by the CDC WONDER database. Deaths due to chronic diseases related to alcohol use or for which alcohol is a contributing factor but not the primary cause of death, such as injuries, certain cancers, or cardiovascular events, were not included in our analyses, potentially underestimating the overall death burden. Since the database suppresses entries with fewer than 10 deaths, data are not available for combined gender, age or race strata as their monthly numbers will be extremely low. This is the case for monthly mortality within 2018–2024 for those aged 15–24. We thus combined the 15–24 and 25–34 age groups into a single 15–34 category to ensure sufficient counts for the Rbeast trend analysis. Since our initial submission, some of the provisional data for 2024 have been updated; changes are minor and do not alter the overall conclusions of this study.

### 4.2. Public health implications

The rise in alcohol-induced mortality and its heterogeneous trends across demographics highlight the need for a better understanding of the socioeconomic factors linked to excessive alcohol consumption and of targeted prevention and treatment efforts, particularly for males, youth, and the AIAN population. Although mortality is lower, the accelerated rise in crude rates among females relative to males is a major cause for concern. Intervention is particularly urgent given that elevated mortality is only one of many negative outcomes associated with excessive alcohol consumption, a crisis which impacts public health, safety, healthcare expenditures, and family stability. Finally, in addition to drug-induced mortality and suicide, alcohol-induced mortality is one of the major contributors to the “deaths of despair” crisis in the United States. All three mortalities affect many socio-economic and demographic groups [25,41,47,48], although those who experience economic and job insecurity, loneliness, and overall feelings of disenfranchisement, are particularly vulnerable. Viewed through this lens, alcohol-induced deaths should be analyzed in conjunction with drug-overdoses and suicides since they may share the same socio-economic drivers and have all been generally rising in recent years. Furthermore, there may be regions where alcohol-related deaths have declined while drug overdose deaths have risen, or vice-versa, due to cultural changes, substance availability, and public awareness. Finally, some individuals may suffer from both alcohol and opioid use disorders, mixing two substances, or exchanging them when one or the other is difficult to obtain. A more holistic approach would thus be very helpful to better quantify and monitor all deaths of despair in the United States.

## Supporting information

S1 Text Supplementary information file, including extra tables and figures. A summary of tables that contain change point statistics and figures of state-level changes in mortalities.
(DOCX)
