# Supplementary material for: Alcohol-induced deaths in the United States across age, race, gender, geography, and the COVID-19 pandemic
Source: PLOS Glob Public Health. 2025 Sep 17;5(9):e0004623. doi: 10.1371/journal.pgph.0004623 (PMC12443242; doi:10.1371/journal.pgph.0004623)
Supplement: S1 Text — A summary of tables that contain change point statistics and figures of state-level changes in mortalities. (DOCX) [file pgph.0004623.s001.docx]

**Alcohol-induced deaths in the United States across age, race, gender, geography, and the COVID-19 pandemic; Supporting Information**

| **Age Group** | **Cause** | **Trend** $T(t_{i-1})$ | **Trend** $T(t_{i})$ | **TCP Jump**  **(pct chg)** | **Credible Interval** |
| --- | --- | --- | --- | --- | --- |
| 15-34 | All causes | $T($Apr '20) = 0.31 | $T($May '20) = 0.40 | May '20 (+28%) | Mar-Jun '20 |
| 15-34 | Mental/  Behavioral | $T($Mar '20) = 0.13 | $T($Apr '20) = 0.19 | Apr '20 (+43%) | Mar-Jun '20 |
| 35-44 | All causes | $T($Apr '20) = 1.48 | $T($May '20) = 1.87 | May '20 (+26%) | Mar-Jul '20 |
| 35-44 | ALD | $T($Apr '20) = 0.75 | $T($May '20) = 1.06 | May '20 (+42%) | Apr-Sep '20 |
| 35-44 | ALD | $T($Jan '23) = 1.06 | $T($Feb '23) = 0.97 | Feb '23 (-9%) | Nov '22 – May '23 |
| 35-44 | Mental/  Behavioral | $T($Mar '20) = 0.70 | $T($Apr '20) = 0.85 | Apr '20 (+22%) | Feb-Jun '20 |
| 45-54 | All causes | $T($Apr '20) = 2.67 | $T($May '20) = 3.33 | May '20 (+24%) | Mar-Jun '20 |
| 45-54 | All causes | $T($Feb '22) = 3.41 | $T($Mar '22) = 3.03 | Mar '22 (-11%) | Jan-Apr '22 |
| 45-54 | ALD | $T($Apr '20) = 1.58 | $T($May '20) = 1.84 | May '20 (+17%) | Mar-Jul '20 |
| 45-54 | ALD | $T($Jan '22) = 2.06 | $T($Feb '22) = 1.94 | Feb '22 (-6%) | Oct '21 - Apr '22 |
| 45-54 | Mental/  Behavioral | $T($Feb '20) = 1.03 | $T($Mar '20) = 1.35 | Mar '20 (+31%) | Feb-May '20 |
| 45-54 | Mental/  Behavioral | $T($Jan '22) = 1.55 | $T($Feb '22) = 1.39 | Feb '22 (-11%) | Jan-May '22 |
| 55-64 | All causes | $T($Feb '20) = 4.14 | $T($Mar '20) = 4.55 | Mar '20 (+10%) | Jan-Jun '20 |
| 55-64 | ALD | $T($Jun '20) = 2.59 | $T($Jul '20) = 2.73 | Jul '20 (+5%) | Dec '19 - Aug '20 |
| 55-64 | Mental/  Behavioral | $T($Jan '20) = 1.57 | $T($Feb '20) = 1.93 | Feb '20 (+23%) | Jan-Apr '20 |
| 65-74 | All causes | $T($Mar '20) = 3.15 | $T($Apr '20) = 3.73 | Apr '20 (+18%) | Feb-May '20 |
| 65-74 | ALD | $T($Mar '20) = 1.96 | $T($Apr '20) = 2.26 | Apr '20 (+16%) | Feb-Jun '20 |
| 65-74 | Mental/  Behavioral | $T($Mar '20) = 1.31 | $T($Apr '20) = 1.49 | Apr '20 (+14%) | Aug '19 - May '20 |
| 75+ | Mental/  Behavioral | $T($Mar '20) = 0.63 | $T($Apr '20) = 0.74 | Apr '20 (+18%) | Feb-Jul '20 |
|  | | | | | |

**Table A.** **TCP jumps for male monthly crude rates stratified by age group and cause of death from 2018 to 2024.** Values are plotted in Fig 4 of the main text. The percent change (pct chg) at month $t_{i}$ is defined as 100 $\text{TCP}(t_{i})/T(t_{i-1})$, where $\text{TCP}(t_{i})= T(t_{i})-T(t_{i-1})$ is the TCP jump. Mortality is highest among those between 55-64. The largest relative increase is for males aged 15-34 (TCP jump +28%, May '20). Deaths due to ALD rose the most for those aged 35-44 (+42%, May '20); deaths due to mental and behavioral issues rose the most for those aged 15-34 (+43%, Apr '20).

| **Age Group** | **Cause** | **Trend** $T(t_{i-1})$ | **Trend** $T(t_{i})$ | **TCP Jump**  **(pct chg)** | **Credible Interval** |
| --- | --- | --- | --- | --- | --- |
| 35-44 | All causes | $T$(Apr '20) = 0.68 | $T($May '20) = 0.88 | May '20 (+28%) | Mar-Jul '20 |
| 35-44 | ALD | $T$(Apr '20) = 0.44 | $T($May '20) = 0.6 | May '20 (+35%) | Mar-Dec '20 |
| 35-44 | Mental/  Behavioral | $T$(Apr '20) = 0.26 | $T($May '20) = 0.31 | May '20 (+20%) | Feb-Oct '20 |
| 45-54 | All causes | $T($Apr '20) = 1.17 | $T($May '20) = 1.41 | May '20 (+21%) | Mar-Jul '20 |
| 45-54 | ALD | $T($May '20) = 0.91 | $T($Jun '20) = 1.05 | Jun '20 (+15%) | Apr-Sep '20 |
| 45-54 | Mental/  Behavioral | $T($Mar '20) = 0.38 | $T($Apr '20) = 0.45 | Apr '20 (+20%) | Feb-Jul '20 |
| 45-54 | Poisoning | $T($May '24) = 0.07 | $T($Jun '24) = 0.00 | Jun '24 (-93%) | May-Jul '24 |
| 55-64 | All causes | $T($Apr '20) = 1.53 | $T($May '20) = 1.83 | May '20 (+20%) | Mar-Jun '20 |
| 55-64 | All causes | $T($Jan '22) = 1.98 | $T($Feb '22) = 1.85 | Feb '22 (-7%) | Jun '21 - May '22 |
| 55-64 | ALD | $T($Apr '20) = 1.04 | $T($May '20) = 1.26 | May '20 (+21%) | Mar-Jun '20 |
| 55-64 | ALD | $T($Jan '22) = 1.42 | $T($Feb '22) = 1.26 | Feb '22 (-12%) | Jan '22 – Mar '22 |
| 55-64 | Mental/  Behavioral | $T($Apr '20) = 0.55 | $T($May '20) = 0.62 | May '20 (+12%) | Feb-Nov '20 |
| 65-74 | All causes | $T($Mar '20) = 0.93 | $T($Apr '20) = 1.08 | Apr '20 (+17%) | Feb '20 – Sep '20 |
| 65-74 | ALD | $T($Jun '20) = 0.71 | $T($Jul '20) = 0.77 | Jul '20 (+8%) | Mar-Dec '20 |
| 65-74 | Mental/  Behavioral | $T($Feb '20) = 0.31 | $T($Mar '20) = 0.37 | Mar '20 (+21%) | Jan-May '20 |

**Table B. TCP jumps for female monthly crude rates stratified by age group and cause of death from 2018 to 2024.** Values are plotted in Fig 4 of the main text. The percent change (pct chg) at month $t_{i}$ is defined as 100 $\text{TCP}(t_{i})/T(t_{i-1})$, where $\text{TCP}(t_{i})= T(t_{i})-T(t_{i-1})$ is the TCP jump. Mortality is highest among those between 55-64. The largest relative increase among females is for those aged 35-44 (TCP jump +28%, May '20). Deaths due to ALD rose the most for those aged 35-44 (+35%, May '20) while deaths due to mental and behavioral issues also rose the most for those aged 65-74 (+21%, Mar '20), followed closely by those aged 35-44 (+20%, May '20) and 45-54 (+20%, Apr '20).

|  | | | | | |
| --- | --- | --- | --- | --- | --- |
| **Race** | **Gender** | **Trend** $T(t_{i-1})$ | **Trend** $T(t_{i})$ | **TCP Jump**  **(pct chg)** | **Credible Interval** |
| AIAN | Male | $T($May '20) = 5.97 | $T($Jun '20) = 8.44 | Jun '20 (+41%) | Apr-Jul '20 |
| AIAN | Male | $T($Dec '20) =8.53 | $T($Jan '21) = 9.34 | Jan '21 (+9%) | Dec '20 - Oct '21 |
| AIAN | Female | $T($Jun '20) = 4.24 | $T($Jul '20) = 5.59 | Jul '20 (+32%) | Apr-Sep '20 |
| AIAN | Female | $T($Jan '22) = 5.52 | $T($Feb '22) = 5.03 | Feb '22 (-9%) | Dec '21 - Apr '22 |
| Black | Male | $T($Apr '20) = 1.13 | $T($May '20) = 1.29 | May '20 (+14%) | Jan '20 - Jul '20 |
| Black | Male | $T($Jan '22) = 1.51 | $T($Feb '22) = 1.25 | Feb '22 (-17%) | Jan-May '22 |
| Black | Female | $T($Apr '20) = 0.40 | $T($May '20) = 0.53 | May '20 (+32%) | Mar-Jun '20 |
| Black | Female | $T($Jan '22) = 0.60 | $T($Feb '22) = 0.48 | Feb '22 (-21%) | Jan-Mar '22 |
| Hispanic | Male | $T($May '20) = 1.37 | $T($Jun '20) = 1.58 | Jun '20 (+16%) | Mar-Nov '20 |
| Mixed race | Male | $T($Apr '20) = 0.34 | $T($May '20) = 0.46 | May '20 (+37%) | Dec '19 - Jul '20 |
| Mixed race | Male | $T($Feb '22) = 0.46 | $T($Mar '22) = 0.40 | Mar '22 (-13%) | Jan '22 - Jul '22 |
| White | Male | $T($Mar '20) = 1.70 | $T($Apr '20) = 2.08 | Apr '20 (+22%) | Mar-May '20 |
| White | Male | $T($Jan '22) = 2.25 | $T($Feb '22) = 2.14 | Feb '22 (-5%) | Dec '21 – Jun '22 |
| White | Female | $T($Mar '20) = 0.72 | $T($Apr '20) = 0.80 | Apr '20 (+12%) | Mar-Jul '20 |

**Table C. TCP jumps for monthly crude rates stratified by races for both genders from 2018 to 2024.** Values are plotted in Fig 5 of the main text. The percent change (pct chg) at month $t_{i}$ is defined as 100 $\text{TCP}(t_{i})/T(t_{i-1})$, where $\text{TCP}(t_{i})= T(t_{i})-T(t_{i-1})$ is the TCP jump. Mortality is highest for the AIAN male and female populations. The largest relative increase is among AIAN males (TCP jump +41% in Jun '20) and among AIAN females (TCP jump + 32%, in Jul '20) and Black females (TCP jump + 32%, May '20). Males exhibited a TCP in early 2020 across all races, except Asian.

| **State** | **Male Rate (2019)** | **Male Rate (2021)** | **Male Rate (2024)** | **Male Pct chg (2019 - 2021)** | **Male Pct chg (2019 - 2024)** |
| --- | --- | --- | --- | --- | --- |
| Hawaii | 9.7 | 14.1 | 9.0 | +44.0% | -7.0% |
| Maryland | 10.3 | 16.0 | 12.4 | +54.0% | +20.0% |
| New Jersey | 11.8 | 12.9 | 9.2 | +9.0% | -22.0% |
| Pennsylvania | 12.0 | 16.1 | 12.6 | +35.0% | +5.0% |
| Louisiana | 12.0 | 16.3 | 13.1 | +36.0% | +9.0% |
| Utah | 12.2 | 16.4 | 13.0 | +35.0% | +7.0% |
| New York | 12.3 | 14.5 | 12.4 | +18.0% | +1.0% |
| Mississippi | 12.5 | 28.2 | 20.1 | +125.0% | +60.0% |
| Alabama | 13.0 | 18.3 | 13.5 | +41.0% | +4.0% |
| Illinois | 13.2 | 18.9 | 16.9 | +43.0% | +28.0% |
| Georgia | 13.4 | 18.2 | 14.8 | +36.0% | +11.0% |
| Virginia | 13.6 | 17.3 | 12.9 | +26.0% | -5.0% |
| Texas | 13.7 | 17.7 | 14.6 | +30.0% | +7.0% |
| Missouri | 14.7 | 22.2 | 17.9 | +50.0% | +21.0% |
| Dist. Columbia | 15.2 | 22.6 | 13.4 | +48.0% | -12.0% |
| Massachusetts | 15.4 | 19.8 | 14.9 | +29.0% | -3.0% |
| Ohio | 15.4 | 22.4 | 17.8 | +45.0% | +15.0% |
| Delaware | 16.1 | 24.4 | 15.2 | +51.0% | -6.0% |
| Arkansas | 16.2 | 20.0 | 16.3 | +23.0% | +1.0% |
| North Carolina | 16.8 | 23.0 | 17.6 | +37.0% | +5.0% |
| Connecticut | 17.1 | 21.9 | 18.8 | +28.0% | +10.0% |
| Kansas | 17.3 | 26.4 | 21.0 | +53.0% | +21.0% |
| Michigan | 17.4 | 24.9 | 20.1 | +43.0% | +15.0% |
| Indiana | 18.0 | 24.4 | 20.8 | +35.0% | +15.0% |
| Florida | 18.1 | 23.4 | 18.2 | +29.0% | +0.0% |
| South Carolina | 18.4 | 28.1 | 21.1 | +52.0% | +15.0% |
| Kentucky | 19.0 | 23.6 | 20.6 | +24.0% | +8.0% |
| Iowa | 19.2 | 28.3 | 24.9 | +48.0% | +30.0% |
| California | 19.4 | 26.7 | 21.2 | +37.0% | +9.0% |
| Nebraska | 19.9 | 24.9 | 23.5 | +25.0% | +18.0% |
| Minnesota | 19.9 | 28.1 | 24.7 | +41.0% | +24.0% |
| Rhode Island | 20.4 | 29.2 | 22.7 | +44.0% | +11.0% |
| Wisconsin | 20.9 | 28.9 | 23.9 | +38.0% | +14.0% |
| Maine | 21.3 | 34.0 | 27.8 | +60.0% | +31.0% |
| Tennessee | 21.3 | 28.9 | 22.9 | +36.0% | +8.0% |
| Washington | 21.4 | 30.1 | 23.6 | +40.0% | +10.0% |
| West Virginia | 21.9 | 28.9 | 20.9 | +32.0% | -4.0% |
| Idaho | 22.1 | 28.2 | 25.7 | +27.0% | +16.0% |
| N. Hampshire | 22.4 | 27.4 | 23.3 | +22.0% | +4.0% |
| Arizona | 24.3 | 35.6 | 26.2 | +46.0% | +8.0% |
| Nevada | 24.7 | 34.3 | 26.2 | +39.0% | +6.0% |
| Oklahoma | 25.3 | 30.7 | 22.4 | +21.0% | -12.0% |
| South Dakota | 25.5 | 53.4 | 43.9 | +109.0% | +72.0% |
| Colorado | 28.6 | 39.8 | 32.1 | +39.0% | +12.0% |
| Alaska | 29.6 | 52.3 | 28.3 | +77.0% | -5.0% |
| Vermont | 29.8 | 34.0 | 27.4 | +14.0% | -8.0% |
| Montana | 30.1 | 44.5 | 34.8 | +48.0% | +16.0% |
| North Dakota | 30.3 | 34.1 | 34.3 | +13.0% | +13.0% |
| Oregon | 30.9 | 41.5 | 33.3 | +34.0% | +8.0% |
| Wyoming | 46.1 | 55.4 | 38.5 | +20.0% | -17.0% |
| New Mexico | 48.9 | 71.0 | 46.9 | +45.0% | -4.0% |

**Table D. Male yearly crude rates in 2019, 2021, and 2024 stratified by state.** Values are plotted in Fig 6 of the main text. The male crude rate is highest in New Mexico for all years. Crude rates increased in all states between 2019 and 2021, with the largest relative increase occurring in Mississippi (+125%), followed by South Dakota (+109%). Crude rates declined in 2024 compared to 2021 peak values in all states, except for North Dakota, where no appreciable changes were recorded, but remained elevated compared to 2019 in 39 states. The largest differential is in South Dakota, where the 2024 crude rate is 72% higher than in 2019, followed by Mississippi (+60%). Of the 13 states where 2024 crude rates were less than in 2019, New Jersey displays the smallest relative increase from 2019 to 2021 (+9%) and experienced the largest overall decline from 2019 to 2024 (-22%).

| **State** | **Female Rate (2019)** | **Female Rate (2021)** | **Female Rate (2024)** | **Female Pct chg (2019 - 2021)** | **Female Pct chg (2019 - 2024)** |
| --- | --- | --- | --- | --- | --- |
| Hawaii | 3.8 | 5.3 | 3.6 | +39.0% | -5.0% |
| Maryland | 4.1 | 4.7 | 4.2 | +15.0% | +2.0% |
| New Jersey | 4.1 | 5.1 | 3.5 | +27.0% | -14.0% |
| Pennsylvania | 4.5 | 6.7 | 5.3 | +47.0% | +17.0% |
| Louisiana | 4.0 | 6.1 | 4.9 | +53.0% | +21.0% |
| Utah | 5.8 | 9.3 | 6.4 | +59.0% | +10.0% |
| New York | 4.2 | 4.9 | 4.1 | +17.0% | -2.0% |
| Mississippi | 3.8 | 8.2 | 7.7 | +113.0% | +99.0% |
| Alabama | 4.7 | 7.4 | 5.7 | +59.0% | +22.0% |
| Illinois | 4.9 | 6.7 | 6.3 | +36.0% | +28.0% |
| Georgia | 4.6 | 7.4 | 5.7 | +60.0% | +23.0% |
| Virginia | 4.6 | 6.3 | 5.6 | +37.0% | +20.0% |
| Texas | 4.7 | 6.3 | 5.3 | +33.0% | +13.0% |
| Missouri | 5.2 | 7.8 | 7.0 | +49.0% | +34.0% |
| Dist. Columbia | - | 8.3 | 5.9 | - | - |
| Massachusetts | 6.3 | 9.2 | 6.9 | +46.0% | +10.0% |
| Ohio | 6.0 | 7.9 | 6.6 | +31.0% | +10.0% |
| Delaware | 6.0 | 7.6 | 8.4 | +27.0% | +42.0% |
| Arkansas | 6.6 | 7.5 | 6.4 | +13.0% | -4.0% |
| North Carolina | 6.0 | 8.3 | 6.2 | +40.0% | +4.0% |
| Connecticut | 6.1 | 8.4 | 7.2 | +39.0% | +18.0% |
| Kansas | 7.0 | 10.0 | 8.0 | +43.0% | +15.0% |
| Michigan | 6.6 | 10.0 | 8.1 | +51.0% | +22.0% |
| Indiana | 5.6 | 8.5 | 7.0 | +51.0% | +25.0% |
| Florida | 7.4 | 9.3 | 7.9 | +26.0% | +6.0% |
| South Carolina | 6.1 | 11.2 | 9.2 | +84.0% | +52.0% |
| Kentucky | 5.9 | 7.1 | 6.4 | +20.0% | +9.0% |
| Iowa | 6.8 | 9.7 | 8.8 | +44.0% | +30.0% |
| California | 7.7 | 10.5 | 8.9 | +36.0% | +15.0% |
| Nebraska | 8.2 | 12.1 | 7.1 | +48.0% | -13.0% |
| Minnesota | 9.5 | 12.6 | 11.5 | +33.0% | +22.0% |
| Rhode Island | 6.3 | 10.6 | 10.2 | +69.0% | +63.0% |
| Wisconsin | 8.9 | 10.9 | 10.7 | +23.0% | +21.0% |
| Maine | 9.9 | 13.9 | 10.0 | +41.0% | +1.0% |
| Tennessee | 6.5 | 9.4 | 8.5 | +44.0% | +30.0% |
| Washington | 11.8 | 15.6 | 13.2 | +33.0% | +12.0% |
| West Virginia | 6.4 | 6.8 | 5.9 | +6.0% | -9.0% |
| Idaho | 10.3 | 15.5 | 12.5 | +50.0% | +21.0% |
| N. Hampshire | 7.6 | 11.6 | 10.7 | +54.0% | +41.0% |
| Arizona | 11.1 | 16.4 | 14.7 | +48.0% | +33.0% |
| Nevada | 10.7 | 17.2 | 12.4 | +60.0% | +16.0% |
| Oklahoma | 8.1 | 11.4 | 8.9 | +40.0% | +9.0% |
| South Dakota | 17.6 | 30.4 | 22.3 | +73.0% | +27.0% |
| Colorado | 12.9 | 18.2 | 15.2 | +41.0% | +17.0% |
| Alaska | 20.6 | 32.7 | 23.0 | +59.0% | +12.0% |
| Vermont | 10.5 | 11.1 | 12.3 | +6.0% | +17.0% |
| Montana | 15.8 | 22.6 | 14.7 | +43.0% | -7.0% |
| North Dakota | 8.3 | 18.3 | 12.0 | +120.0% | +45.0% |
| Oregon | 13.7 | 17.7 | 18.6 | +29.0% | +36.0% |
| Wyoming | 14.8 | 23.4 | 25.6 | +58.0% | +73.0% |
| New Mexico | 21.0 | 32.4 | 20.8 | +54.0% | -1.0% |

**Table E. Female yearly crude rates in 2019, 2021, and 2024 stratified by state.** Values are plotted in Fig 7 of the main text. New Mexico, Alaska, and Wyoming are states with the largest crude rates in 2019, 2021, and 2024, respectively. In 2021, crude rates increased in all states compared to 2019; the largest relative increase occurred in North Dakota (+120%), followed by Mississippi (+113%). In Mississippi, however, crude rates peaked in 2022, corresponding to a 151% increase between 2019 and 2022. By 2024, crude rates had decreased in most states, and in 8 of them, they were below their 2019 values. Crude rates remained particularly elevated in Mississippi, where crude rates in 2024 were still 99% higher than in 2019. Of the 8 states where 2024 crude rates were less than in 2019, New Jersey exhibited the largest decrease (-14%). In 2019, the crude rate for the District of Columbia could not be determined due to low female fatalities.

**Fig A. Percent change in yearly crude rates of mortality due to alcoholic liver disease (ALD), by gender and state, between 2019 and 2021, and between 2019 and 2024.** From 2019 to 2021, ALD crude rates increased for males and females in all states except West Virginia, where the crude rate decreased by 4% among females. The highest relative increases among males were recorded in Mississippi (+164%), South Dakota (+129%), and Alaska (+115%). Among females, the largest relative increases were recorded in Mississippi (+182%), Utah (+104%), and South Carolina (+95%). Mississippi had the largest relative increase in the ALD crude rate for both genders (males: +164%, females: +182%). By 2024, crude rates remained higher than their 2019 levels in 39 states for males and 42 states for females. These results suggest that ALD deaths impacted females more than males.

**Fig. B. Percent change in yearly crude rates of mortality due to mental and behavioral disorders from alcohol use, by gender and state, between 2019 and 2021, and between 2019 and 2024.** States where crude rates were unavailable are shown in gray. From 2019 to 2021, crude rates increased among males in all states. Among females, they increased in all states for which data were available, except for New Jersey (-5%) and Maryland (-7%). By 2024, crude rates remained higher than their 2019 levels in 38 states for males and in 34 for females. These results suggest that deaths due to alcohol-induced mental and behavioral disorders impacted both genders.

**Fig C. Percent change in yearly crude rates of mortality due to alcohol poisoning, by gender and state, between 2019 and 2021, and between 2019 and 2024.** States where crude rates were unavailable are shown in gray. From 2019 to 2021, the largest relative increases in crude rates among males occurred in Maine (+74%), South Carolina (+70%), and Missouri (+65%). Only five states had sufficient female mortality to evaluate crude rates in both 2019 and 2024. For these, relative increases were observed in Colorado (+10%), Texas (+6%), and California (+6%), whereas New Mexico and Florida reported decreasing crude rates. Between 2019 and 2024, crude rates for alcohol-induced poisoning declined for both genders in all states where data were available.
